# Development and validation of a nomogram for predicting metabolic-associated fatty liver disease in the Chinese physical examination population

**DOI:** 10.1186/s12944-023-01850-y

**Published:** 2023-06-29

**Authors:** Bingqian Zhou, Ni Gong, Xinjuan Huang, Jingchi Zhu, Chunxiang Qin, Qingnan He

**Affiliations:** 1 Department of Health Management Center, The Third Xiangya Hospital, Central South University, Changsha, 410013 China; 2 Xiangya Nursing School, Central South University, Changsha, 410013 China; 3 Jishou University School of Medicine, Jishou, 416000 China

**Keywords:** Nomogram, MAFLD, Risk prediction, Physical examination

## Abstract

**Aim:**

We aim to develop and validate a nomogram including readily available clinical and laboratory indicators to predict the risk of metabolic-associated fatty liver disease (MAFLD) in the Chinese physical examination population.

**Methods:**

The annual physical examination data of Chinese adults from 2016 to 2020 were retrospectively analyzed. We extracted the clinical data of 138 664 subjects and randomized participants to the development and validation groups (7:3). Significant predictors associated with MAFLD were identified by using univariate and random forest analyses, and a nomogram was constructed to predict the risk of MAFLD based on a Lasso logistic model. Receiver operating characteristic curve analysis, calibration curves, and decision curve analysis were used to verify the discrimination, calibration, and clinical practicability of the nomogram, respectively.

**Results:**

Ten variables were selected to establish the nomogram for predicting MAFLD risk: sex, age, waist circumference (WC), uric acid (UA), body mass index (BMI), waist-to-hip ratio (WHR), systolic blood pressure (SBP), fasting plasma glucose (FPG), triglycerides (TG), and alanine aminotransferase (ALT). The nomogram built on the nonoverfitting multivariable model showed good prediction of discrimination (AUC 0.914, 95% CI: 0.911–0.917), calibration, and clinical utility.

**Conclusions:**

This nomogram can be used as a quick screening tool to assess MAFLD risk and identify individuals at high risk of MAFLD, thus contributing to the improved management of MAFLD.

**Supplementary Information:**

The online version contains supplementary material available at 10.1186/s12944-023-01850-y.

## Introduction

Metabolic-associated fatty liver disease (MAFLD), a multisystem metabolic disease involving the liver, is an update of nonalcoholic fatty liver disease (NAFLD) and is notable for its redefinition of diagnostic conditions and its emphasis on metabolic factors while considering nonalcoholic factors [1]. MAFLD can not only progress to steatohepatitis, liver cirrhosis, and hepatocellular carcinoma [2] but also increase the occurrence and development of extrahepatic diseases, such as cardiovascular and chronic kidney diseases [3]. At present, MAFLD affects more than one-third of the global population, showing a trend of annual increase and rejuvenation [4, 5]. Meanwhile, in China, between 29 and 46% of the population has MAFLD, which has become the most prevalent chronic liver disease, thus seriously aggravating the medical and economic burden on affected individuals and all societies [6, 7].

MAFLD has an implicit pathogenesis and no specific clinical symptoms in the early stages and is therefore easily ignored. Early detection and management are crucial to prevent the progression of MAFLD. Among existing diagnostic tools, liver biopsy is the gold standard for the diagnosis of MAFLD but is unsuitable for routine screening because it is invasive and expensive [8]. Although ultrasonography is noninvasive, it may not be routinely performed in primary or secondary medical centers [9]. Other imaging tests are too expensive for conducting mass screening effectively. Moreover, some factors related to metabolic dysfunction in the new diagnostic criteria may not be routinely measured owing to the complexity and technicality associated with biomarker measurements and diagnostic equipment, such as the homeostasis model assessment of insulin resistance index (HOMA-IR), plasma high-sensitivity C-reactive protein level (Hs-CRP), glycated hemoglobin (HbA1c), and 2-h postload glucose. This situation limits the application of MAFLD diagnostic criteria by healthcare providers. Therefore, developing a simple, noninvasive, and practical MAFLD prediction model for the rapid screening of MAFLD is particularly necessary. Moreover, the screening tool should be widely applied for the early detection of MAFLD in primary, secondary, and tertiary medical centers.

Some simple screening tools for NAFLD based on demographics, laboratory factors, and anthropometrics have emerged [10–13] (e.g., fatty liver index, NAFLD liver fat score, and the hepatic steatosis index). However, these screening tools are not applicable to the newly defined condition of MAFLD. A nomogram to predict the risk of MAFLD in overweight and obese people has recently been developed but is suitable only for those with body weight index (BMI) ≥ 24 and male waist circumference (WC) ≥ 90 cm or female WC ≥ 80 cm [14]. Another nonimaging-assisted nomogram established in a large United States (US) population could screen for MAFLD well but has unclear applicability to the Chinese population [15]. Therefore, MAFLD screening tools that can be easily used in the Chinese general population have not yet been developed.

Nomograms have been utilized widely to predict the risks of various diseases [16]. They are graphical prediction tools that visually and intuitively quantify the risk of events on the basis of various predictors [17]. Therefore, we aimed to develop and validate a nomogram for MAFLD screening and MAFLD risk classification in the general population based on routine indicators associated with MAFLD during physical examination.

## Materials and methods

### Participants

The study was carried out on the basis of a physical examination survey among individuals who underwent annual physical examinations at the Health Management Center of the Third Xiangya Hospital of Central South University in Hunan Province, China, between 2016 and 2020 (although all participants were from one medical facility, they were from different provinces of China). This institution is a tertiary medical center with a high ultrasound completion rate. A total of 207,663 individuals who underwent physical examinations between 2016 and 2020 and who were aged 18–79 years old were included in this study **(**Supplementary Fig. 1**)**. The enrollment was limited to participants with complete records of demographic, anthropometric, blood biochemical indicators, and lifestyle information, as well as the results of hepatic ultrasonography examination.

### Data collection

Predictor variables were chosen on the basis of their clinical importance and evidence related to MAFLD. The collected data included demographic information (sex, age, education, marriage, family history of hypertension, and/or diabetes), anthropometric parameters (BMI, WC, waist-to-hip ratio [WHR], systolic blood pressure [SBP], and diastolic blood pressure [DBP]), blood biochemical indicators (alanine aminotransferase [ALT], uric acid [UA], fasting plasma glucose [FPG], total cholesterol [TC], triglyceride [TG], high-density lipoprotein cholesterol [HDL-C], and low-density lipoprotein cholesterol [LDL-C]), and self-reported lifestyles (dietary preference, smoking status, drinking status, physical activity, and sleep duration). In total, 22 variables were collected.

The quality of data collection is controlled by the following procedures. Blood pressure, including SBP and DBP, was measured on the right arm with the participants in a seated position after 5 min of rest. Blood biochemical measurements were performed in the morning on an empty stomach in accordance with standard procedures. Lifestyle-related information was collected by trained clinicians. Outliers and missing values were corrected and added by rechecking the original data in the data management system.

In this study, the same protocol was followed as shown in Transparent Reporting of a Multivariable Predictive Model for Individual Prognosis or Diagnosis [18].

### Definition and assessment

The height and weight of each subject were measured on digital scales to the nearest 0.1 cm and 0.1 kg, respectively. BMI was calculated by weight (kg) divided by the square of height (m^2^). The WC was calculated as the horizontal girth through the navel center. Hip circumference (HC) was defined as the perimeter surrounding the widest part of the buttocks at the axial plane. WHR is calculated by the ratio of WC (cm) to HC (cm).

This study categorized BMI into four groups (underweight: < 18.50 kg/m^2^, normal: 18.50–22.99 kg/m^2^, overweight: 23.00–24.99 kg/m^2^, obese: ≥ 25.00 kg/m^2^) on the basis of the BMI criteria for Asians formulated by the WHO [19]. In our study, the diagnostic criteria for diabetes were or under antidiabetes treatment or self-reported diabetes, and prediabetes was defined as FPG between 5.6 and 6.9 mmol/L (impaired fasting glucose) [20]; SBP of 130 mmHg, DBP of 85 mmHg, being on antihypertensive therapy, or self-reported hypertension were used as diagnostic criteria for hypertension; and abnormal WC was defined as WC ≥ 90 cm for men and WC ≥ 80 cm for women [2]. Abnormal WHR was defined as WHR ≥ 0.90 for men and WHR ≥ 0.85 for women. Hyperuricemia was defined as UA > 420 μmol/L [21]. Elevated liver enzymes were defined as ALT > 40 IU/L [22]. Dyslipidemia was defined as follows: TC ≥ 5.2 mmol/L; LDL-C ≥ 3.4 mmol/L; HDL-C < 1 mmol/L in men and < 1.3 mmol/L in women; and TG ≥ 1.7 mmol/L [23].

Hepatic ultrasound examination was conducted by trained ultrasonographers. Ultrasound diagnosis of hepatic steatosis is based on the presence of hepatic and renal echogenic contrast, liver parenchymal brightness, deep attenuation, and vascular blurring [24]. In reference to the latest MAFLD criteria described by Eslam et al. [1], the diagnosis of MAFLD in this study is based on ultrasonically confirmed steatosis of the liver and one of the following three criteria: overweight or obesity (defined as BMI > 23 kg/m^2^ in Asians), presence of type 2 diabetes mellitus, or evidence of metabolic dysregulation. Metabolic dysregulation was defined as the presence of ≥ 2 of the following [1]: (i) WC ≥ 90/80 cm (Asian cutoff) in men and women, respectively; (ii) blood pressure ≥ 130/85 mmHg or specific drug treatment; (iii) TG ≥ 1.7 mmol/L or specific drug treatment; (iv) HDL-C < 1.0 mmol/L in men and < 1.3 mmol/L in women; (v) prediabetes (i.e., FPG of 5.6 to 6.9 mmol/L or HbA1c of 5.7% to 6.4% or 2-h postload glucose level of 7.8 to 11.0 mmol); (vi) HOMA-IR score ≥ 2.5; and (vii) Hs-CRP level > 2 mg/L.

### Statistical analyses

There was a 7:3 ratio of subjects randomly divided into development and validation datasets for the construction and validation of the nomogram. To develop the model, the development dataset was used, and the validation dataset was used to validate it. The comparability between the two datasets was then evaluated. Categorical variables were presented as numbers (percentages) and compared by using the χ2 test. To identify the potential predictors of MAFLD, two statistical methods were used: univariate regression analysis and the random forest algorithm [25]. We used random forest analysis to calculate the mean decreased Gini (MDG) of each independent variable in this study, which could be used as a measure of this variable's contribution to the risk of MAFLD and explain how the independent and dependent variables are related. In the follow-up analysis, we selected the variables that reached statistical significance in univariate regression analysis (*P* < 0.05) and the top 50% of the random forest MDG. A multivariable logistic regression model was then based on the statistically significant variables identified during these procedures. To ensure that the multivariable logistic regression model was not overfitting, least absolute shrinkage and selection operator regression (LASSO) was performed to eliminate factors with high correlation. Ultimately, a nomogram based on the multivariate model composed of the optimal features was developed to predict the risk of MAFLD. The receiver operating characteristic curve (ROC) was also applied to evaluate discrimination performance, and the AUC (area under the ROC curve) was greater than 0.70, reflecting the high performance of this nomogram [26]. The concordance between the practical results and the predicted probabilities was measured by calibration curves. The clinical practicability of the nomogram was evaluated by decision curve analysis (DCA). The DCA method is used to evaluate and compare predictive models and calculate the net benefits against threshold probabilities [27].

Statistical analysis was performed with R software version 4.2.2 and SPSS version 24.0. A two-sided *P* value < 0.05 was considered statistically significant.

## Results

### Characteristics of subjects

After rigorous screening, 138 664 participants, including 77 951 men and 60 713 women, were finally enrolled. Supplementary Fig. 1 shows the process of selecting subjects. By using the novel MAFLD diagnostic criteria, the MAFLD prevalence was found to be 39.55% (men: 53.39%, women: 21.77%, *P* < 0.001). In our study, participants’ data were randomly assigned 7:3 between the development dataset (*n* = 97 066) and the validation dataset (*n* = 41 598). The prevalence of MAFLD between the two datasets was not significantly different (development dataset: 39.55%, validation dataset: 39.55%, *P* = 0.998). The characteristics of the two datasets are shown in Table 1.

**Table 1:** Characteristics of participants in the development and validation datasets

Variables	Development dataset			Validation dataset		
	n	MAFLD	Non-MAFLD	n	MAFLD	Non-MAFLD
		n (%)	n (%)		n (%)	n (%)
**Sex**						
Female	42,634	9322 (21.87)	33,312 (78.13)	18,079	3898 (21.56)	14,181 (78.44)
Male	54,432	29,066 (53.40)	25,366 (46.60)	23,519	12,553 (53.37)	10,966 (46.63)
**Age**						
18–29	13,567	2566 (18.91)	11,001 (81.09)	6026	1135 (18.84)	4891 (81.16)
30–44	37,410	13,393 (35.80)	24,017 (64.20)	15,977	5781 (36.18)	10,196 (63.82)
45–59	33,514	16,437 (49.05)	17,077 (50.95)	14,290	6993 (48.94)	7297 (51.06)
60–79	12,575	5992 (47.65)	6583 (52.35)	5305	2542 (47.92)	2763 (52.08)
**BMI, kg/m**^**2**^						
< 18.50	3570	9 (0.25)	3561 (99.75)	1524	5 (0.33)	1519 (99.67)
18.50–22.99	34,361	1963 (5.71)	32,398 (94.29)	14,621	810 (5.54)	13,811 (94.46)
23.00–24.99	23,187	9088 (39.19)	14,099 (60.81)	9834	3801 (38.65)	6033 (61.35)
≥ 25.00	35,948	27,328 (76.02)	8620 (23.98)	15,619	11,835 (75.77)	3784 (24.23)
^**a**^**WC**						
Normal	64,706	14,555 (22.49)	50,151 (77.51)	27,730	6246 (22.52)	21,484 (77.48)
Abnormal	32,360	23,833 (73.65)	8527 (26.35)	13,868	10,205 (73.59)	3663 (26.41)
^**b**^**WHR**						
Normal	52,194	9160 (17.55)	43,034 (82.45)	22,337	3946 (17.67)	18,391 (82.33)
Abnormal	44,872	29,228 (65.14)	15,644 (34.86)	19,261	12,505 (64.92)	6756 (35.08)
**SBP, mmHg**						
< 130	68,070	21,415 (31.46)	46,655 (68.54)	29,253	9173 (31.36)	20,080 (68.64)
≥ 130	28,996	16,973 (58.54)	12,023 (41.46)	12,345	7278 (58.96)	5067 (41.04)
**DBP, mmHg**						
< 85	79,126	26,704 (33.75)	52,422 (66.25)	33,869	11,380 (33.60)	22,489 (66.40)
≥ 85	17,940	11,684 (65.13)	6256 (34.87)	7729	5071 (65.61)	2658 (34.39)
**ALT, U/L**						
≤ 40	82,254	27,422 (33.34)	54,832 (66.66)	35,194	11,742 (33.36)	23,452 (66.64)
> 40	14,812	10,966 (74.03)	3846 (25.97)	6404	4709 (73.53)	1695 (26.47)
**UA, μmol/L**						
≤ 420	80,451	26,871 (33.40)	53,580 (66.60)	34,523	11,538 (33.42)	22,985 (66.58)
> 420	16,615	11,517 (69.32)	5098 (30.68)	7075	4913 (69.44)	2162 (30.56)
**FPG, mmol/L**						
< 5.6	68,576	21,118 (30.80)	47,458 (69.20)	29,453	9078 (30.82)	20,375 (69.18)
≥ 5.6	28,490	17,270 (60.62)	11,220 (39.38)	12,145	7373 (60.71)	4772 (39.29)
**TC, mmol/L**						
< 5.2	59,166	19,282 (32.59)	39,884 (67.41)	25,518	8350 (32.72)	17,168 (67.28)
≥ 5.2	37,900	19,106 (50.41)	18,794 (49.59)	16,080	8101 (50.38)	7979 (49.62)
**TG, mmol/L**						
< 1.7	62,171	14,364 (23.10)	47,807 (76.90)	26,694	6184 (23.17)	20,510 (76.83)
≥ 1.7	34,895	24,024 (68.85)	10,871 (31.15)	14,904	10,267 (68.89)	4637 (31.11)
**HDL-C**						
Normal	77,992	27,881 (35.75)	50,111 (64.25)	33,492	12,031 (35.92)	21,461 (64.08)
Abnormal	19,074	10,507 (55.09)	8567 (44.91)	8106	4420 (54.53)	3686 (45.47)
**LDL-C, mmol/L**						
< 3.4	74,448	27,821 (37.37)	46,627 (62.63)	31,896	11,891 (37.28)	20,005 (62.72)
≥ 3.4	22,618	10,567 (46.72)	12,051 (53.28)	9702	4560 (47.00)	5142 (53.00)
**Education**						
Junior high school and below	13,585	5662 (41.68)	7923 (58.32)	5860	2463 (42.03)	3397 (57.97)
Senior high school	18,101	7772 (42.94)	10,329 (57.06)	7682	3279 (42.68)	4403 (57.32)
College and above	65,380	24,954 (38.17)	40,426 (61.83)	28,056	10,709 (38.17)	17,347 (61.83)
**Marriage**						
Married	83,653	35,190 (42.07)	48,463 (57.93)	35,817	15,063 (42.06)	20,754 (57.94)
Other	13,413	3198 (23.84)	10,215 (76.16)	5781	1388 (24.01)	4393 (75.99)
**Family history**						
No	69,508	26,696 (38.41)	42,812 (61.59)	29,725	11,458 (38.55)	18,267 (61.45)
Yes	27,558	11,692 (42.43)	15,866 (57.57)	11,873	4993 (42.05)	6880 (57.95)
**Dietary preference**						
Other diets	55,885	24,084 (43.10)	31,801 (56.90)	24,203	10,481 (43.30)	13,722 (56.70)
Light diet	41,181	14,304 (34.73)	26,877 (65.27)	17,395	5970 (34.32)	11,425 (65.68)
**Smoking status**						
Nonsmoking	65,771	21,952 (33.38)	43,819 (66.62)	27,929	9360 (33.51)	18,569 (66.49)
^c^Quit smoking	1854	979 (52.80)	875 (47.20)	798	435 (54.51)	363 (45.49)
Passive smoking	4115	1765 (42.89)	2350 (57.11)	1851	802 (43.33)	1049 (56.67)
Smoking	25,326	13,692 (54.06)	11,634 (45.94)	11,020	5854 (53.12)	5166 (46.88)
**Drinking status**						
No drinking	65,610	21,688 (33.06)	43,922 (66.94)	27,990	9195 (32.85)	18,795 (67.15)
Drinking	30,191	16,081 (53.26)	14,110 (46.74)	13,026	6990 (53.66)	6036 (46.34)
^d^Quit drinking	1265	619 (48.93)	646 (51.07)	582	266 (45.70)	316 (54.30)
**Physical activity**						
No	36,783	13,914 (37.83)	22,869 (62.17)	16,026	6072 (37.89)	9954 (62.11)
Yes	60,283	24,474 (40.60)	35,809 (59.40)	25,572	10,379 (40.59)	15,193 (59.41)
**Sleep duration**						
5–7 h	60,714	24,756 (40.77)	35,958 (59.23)	26,090	10,667 (40.89)	15,423 (59.11)
< 5 h	8789	3851 (43.82)	4938 (56.18)	3754	1634 (43.53)	2120 (56.47)
> 7 h	27,563	9781 (35.49)	17,782 (64.51)	11,754	4150 (35.31)	7604 (64.69)

^a^Abnormal is defined as WC ≥ 90 cm for men and WC ≥ 80 cm for women

^b^Abnormal is defined as WHR ≥ 0.90 for men and WHR ≥ 0.85 for women

^c^Quit smoking is defined as having stopped smoking for 1 year or more

^d^Quit drinking is defined as having stopped drinking for 1 year or more

### Identifying predictors and constructing a nomogram for MAFLD

Table 2 shows the results of univariate logistic regression analysis and random forest for MAFLD. The default value of Ntree is 500; when mtry = 3 and ntree = 500, out-of-bag samples had the lowest estimation error rate (OOB = 16.37%). All variables were statistically significant in univariate logistic regression analysis. However, 11 variables failed to achieve a high MDG in random forest analysis. The other variables (BMI, WC, WHR, TGs, sex, ALT, FPG, age, UA, SBP, and smoking status) obtained relatively high MDGs (top 50%) in addition to producing significant results in univariate analysis (*P* < 0.05). Therefore, further multivariate modeling was conducted using these 11 variables.

**Table 2:** Univariate regression and random forest results for the development dataset

Variables	Univariate logistic regression		Random forest
	Odds ratio (95%CI)	*P* value	MDG
**BMI, kg/m**^**2**^			7212.068
18.50–22.99	Ref		
< 18.50	0.05 (0.03–0.09)	< 0.001	
23.00–24.99	10.35 (9.82–10.91)	< 0.001	
≥ 25.00	51.75 (49.17–54.48)	< 0.001	
**WC**			3864.336
Normal	Ref		
Abnormal	9.71(9.41–10.01)	< 0.001	
**WHR**			3449.132
Normal	Ref		
Abnormal	8.74(8.48–9.00)	< 0.001	
**TG, mmol/L**			3084.218
< 1.7	Ref		
≥ 1.7	7.39(7.17–7.61)	< 0.001	
**Sex**			1417.176
Female	Ref		
Male	4.08(3.97–4.20)	< 0.001	
**ALT, U/L**			1227.385
≤ 40	Ref		
> 40	5.55(5.34–5.77)	< 0.001	
**FPG, mmol/L**			1012.864
< 5.6	Ref		
≥ 5.6	3.50(3.40–3.60)	< 0.001	
**Age**			989.135
18–29	Ref		
30–44	2.41(2.29–2.52)	< 0.001	
45–59	4.16(3.97–4.36)	< 0.001	
60–79	3.92(3.71–4.14)	< 0.001	
**UA, μmol/L**			920.927
≤ 420	Ref		
> 420	4.52(4.36–4.69)	< 0.001	
**SBP, mmHg**			658.973
< 130	Ref		
≥ 130	3.10(3.01–3.18)	< 0.001	
**Smoking status**			655.454
Non–smoking	Ref		
Quit smoking	2.25(2.05–2.47)	< 0.001	
Passive smoking	1.55(1.45–1.65)	< 0.001	
Smoking	2.34(2.27–2.41)	< 0.001	
**DBP, mmHg**			617.081
< 85	Ref		
≥ 85	3.69 (3.56–3.81)	< 0.001	
**Education**			609.423
Junior high school and below	Ref		
Senior high school	1.05(1.00–1.10)	0.036	
College and above	0.86(0.83–0.89)	< 0.001	
**Sleep duration**			606.523
5–7 h	Ref		
< 5 h	1.17(1.11–1.22)	< 0.001	
> 7 h	0.80(0.78–0.83)	< 0.001	
**Drinking status**			523.462
No drinking	Ref		
Drinking	2.33(2.27–2.40)	< 0.001	
Quit drinking	1.83(1.64–2.04)	< 0.001	
**HDL–C**			505.78
Normal	Ref		
Abnormal	2.19(2.12–2.27)	< 0.001	
**TC, mmol/L**			397.515
< 5.2	Ref		
≥ 5.2	2.10(2.04–2.15)	< 0.001	
**Dietary preference**			360.209
Other diets	Ref		
Light diet	0.69(0.68–0.71)	< 0.001	
**Physical activity**			359.282
No	Ref		
Yes	1.14(1.11–1.17)	< 0.001	
**Family history**			349.978
No	Ref		
Yes	1.17(1.14–1.20)	< 0.001	
**LDL–C, mmol/L**			296.359
< 3.4	Ref		
≥ 3.4	1.47(1.42–1.51)	< 0.001	
**Marriage**			253.486
Married	Ref		
Other	0.43(0.41–0.45)	< 0.001	

*MDG* Mean decrease Gini, *Ref* Reference

**Fig. 1:**
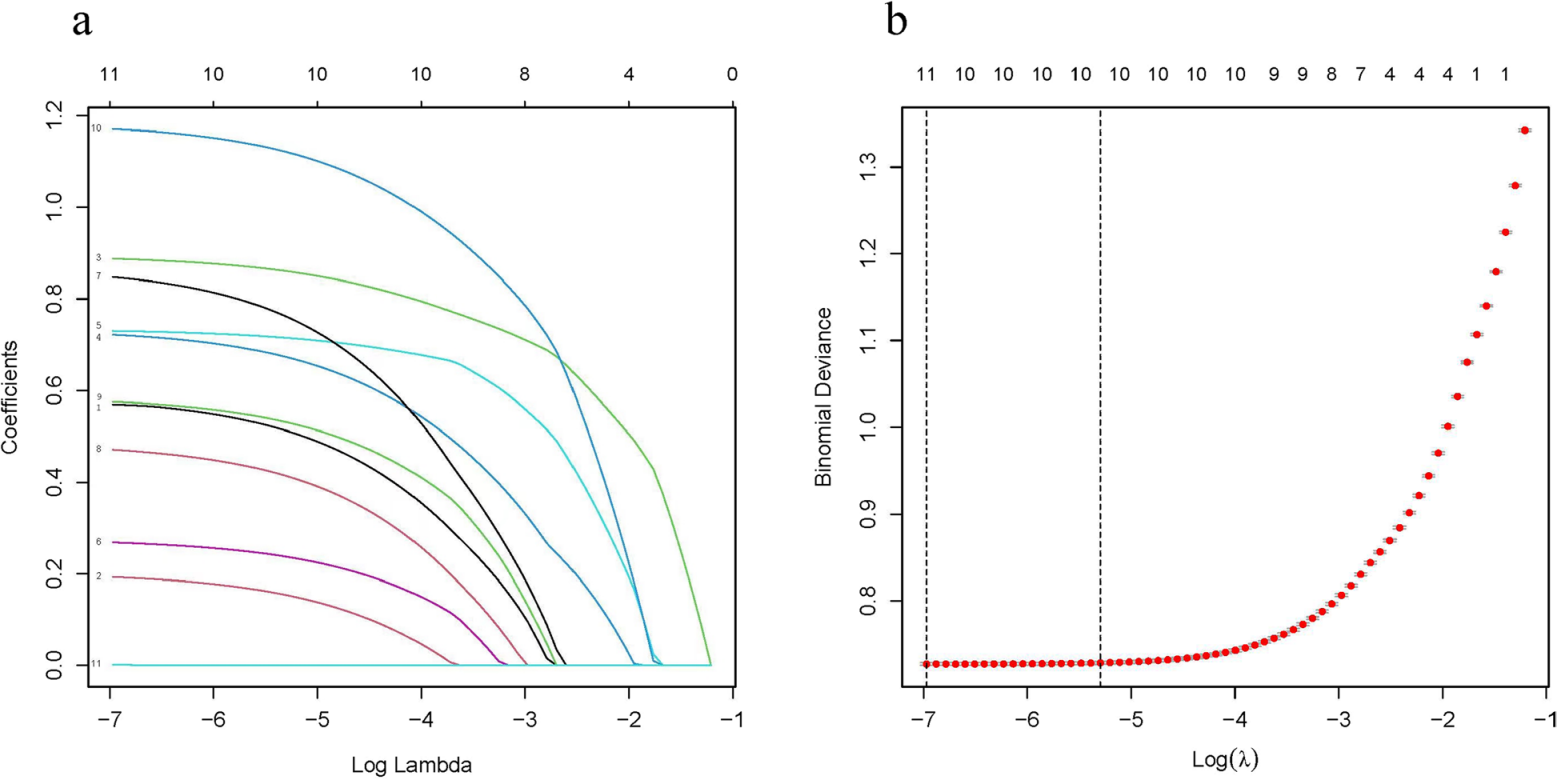
Variable filtering of LASSO regression. Note: **a** LASSO coefficient profile for 11 variables. **b** The selection of the optimal lambda parameter in the LASSO model. To avoid overfitting, LASSO regression suggested including 10 variables (λ = 0.007, log[λ] =  − 5.00)

The modeling process of LASSO regression is shown in Fig. 1a, b. Among the 11 variables (BMI, WC, WHR, TGs, sex, ALT, FPG, age, UA, SBP, and smoking status), 10 independent predictors in the development dataset were identified by the nonzero coefficients in LASSO regression, and the optimal parameter (lambda) selection in the LASSO model was tenfold cross-validated by the minimum criteria. Then, multivariate logistic regression modeling was conducted using the 10 potential risk factors (Table 3). The results showed that BMI ≥ 23.00 kg/m^2^, abnormal WC and WHR, TGs ≥ 1.7 mmol/L, male sex, ALT > 40 U/L, FPG ≥ 5.6 mmol/L, middle age and older age, UA > 420 μmol/L, and SBP ≥ 130 mmHg were independent risk factors for MAFLD.

**Table 3:** Multivariate logistic regression model for MAFLD

Variables	*β*	*S.E*	Odds ratio (95%CI)	*P* value	Score
**BMI, kg/m**^**2**^					
18.50–22.99			Ref		45
< 18.50	–2.18	0.30	0.11(0.06–0.20)	< 0.001	0
23.00–24.99	1.68	0.03	5.39(5.09–5.71)	< 0.001	81
≥ 25.00	2.62	0.03	13.67(12.86–14.54)	< 0.001	100
**WC**					
Normal			Ref		0
Abnormal	0.72	0.03	2.05(1.95–2.16)	< 0.001	15
**WHR**					
Normal			Ref		0
Abnormal	0.70	0.02	2.01(1.93–2.10)	< 0.001	15
**TG, mmol/L**					
< 1.7			Ref		0
≥ 1.7	1.15	0.02	3.16(3.04–3.28)	< 0.001	24
**Sex**					
Female			Ref		0
Male	0.58	0.02	1.78(1.70–1.86)	< 0.001	12
**ALT, U/L**					
≤ 40			Ref		0
> 40	0.87	0.03	2.39(2.26–2.52)	< 0.001	18
**FPG, mmol/L**					
< 5.6			Ref		0
≥ 5.6	0.58	0.02	1.79(1.72–1.87)	< 0.001	12
**Age**					
18–29			Ref		0
30–44	0.42	0.04	1.52(1.42–1.63)	< 0.001	9
45–59	0.69	0.04	2.00(1.86–2.15)	< 0.001	14
60–79	0.61	0.04	1.84(1.69–2.00)	< 0.001	13
**UA, μmol/L**					
≤ 420			Ref		0
> 420	0.50	0.03	1.65(1.57–1.73)	< 0.001	10
**SBP, mmHg**					
< 130			Ref		0
≥ 130	0.29	0.02	1.34(1.29–1.40)	< 0.001	6

*Β* Regression coefficient, *Ref* Reference

**Fig. 2:**
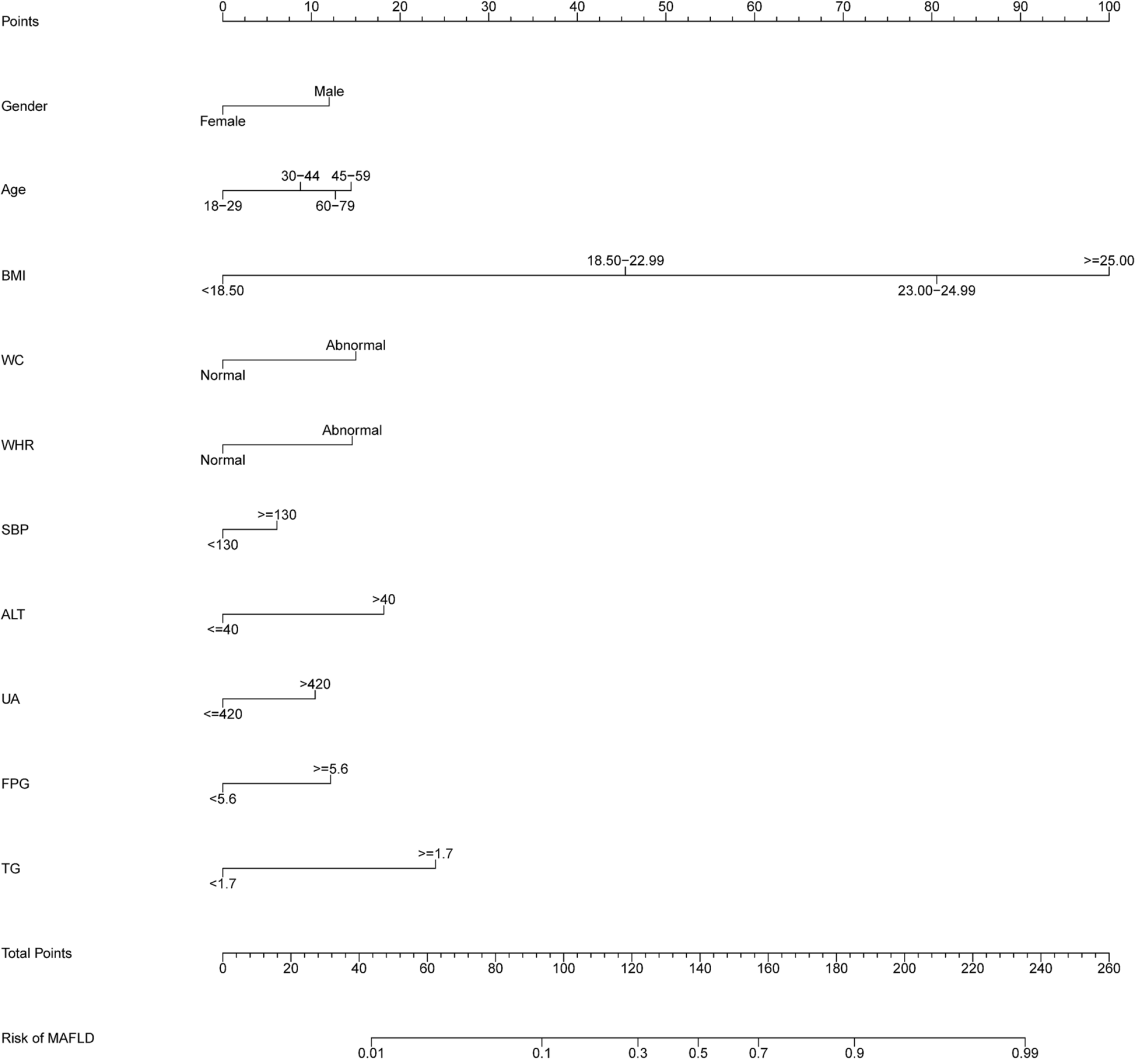
Nomogram for predicting the risk of MAFLD in the physical examination population. Note: When using the nomogram, the corresponding points for each variable were added to obtain the total points, and a vertical line was drawn from the total points axis to the risk of MAFLD axis to obtain the predicted risk value

In accordance with the results of the multivariable logistic regression model, the nomogram for MAFLD was developed on the basis of the 10 risk factors (Fig. 2). To improve the clinical utility of the nomogram, we converted the calculation of risk levels into a prediction table (Supplementary Table 1).

### Discrimination and calibration

After constructing the model using the development dataset (*n* = 97 066), the validation dataset (*n* = 41 598) was used to verify the predictive ability of the model. The AUC of the model was 0.915 (95% CI 0.913–0.916) in the development dataset and 0.914 (95% CI 0.911–0.917) in the validation dataset, indicating good discrimination (Supplementary Fig. 2). For the development dataset, the sensitivity was 0.804, the specificity was 0.863, and the cutoff point was -0.463. For the validation dataset, the sensitivity was 0.787, and the specificity was 0.878, with a cutoff of -0.677. The calibration plot of the current MAFLD rate revealed that the development dataset was similar to the validation dataset, with a slight overestimation of the MAFLD probability between 0.08 and 0.66 and an underestimation above 0.72. Nevertheless, the overall calibration ability was good (Supplementary Fig. 3).

### Clinical practicality

The clinical practicality of the developed nomogram model was evaluated with decision curves (Supplementary Fig. 4). On the basis of decision curve analysis, the threshold probability was ≤ 95% in the development dataset and ≤ 90% in the validation dataset. In other words, when the predicted risk is ≤ 90%, further diagnosis is beneficial. When the predicted risk value is greater than 90%, MAFLD diagnosis has no benefit. In brief, the MAFLD prediction nomogram presented more net benefit than “all individuals with MAFLD” or “no individuals with MAFLD”. As a result, the risk of MAFLD could be classified as low (< 90%) or high (> 90%) in accordance with the developed nomogram.

## Discussion

With the increase in public health awareness, physical examination has become the main way through which people engage in health self-management. In consideration of the background that most patients with MAFLD are diagnosed incidentally during physical examinations and the lack of predictive tools for the large-scale screening of MAFLD risk in the Chinese general population [28], we established and validated a nomogram for predicting MAFLD risk based on real-world large-scale physical examination data by combining classical regression analysis and a random forest algorithm to identify the most significant predictors of MAFLD. The nomogram aims to enable mass screening for MAFLD in primary, secondary, and tertiary care centers by using easily available indicators for the early detection, diagnosis, and intervention of people at risk of MAFLD. Our results showed that our prediction model has good performance in terms of discrimination, calibration, and clinical practicality.

In our study, candidate variables were limited to easily available indicators in the construction of the model for MAFLD prediction. This approach contributed to enhancing the clinical utility of our nomogram. Furthermore, combining classical regression analysis methods and the random forest algorithm guaranteed that we obtained the best combination of predictors. The use of LASSO regression to ensure that multivariate logistic regressions were not overfitted ensured the objectivity of the variables in the model. The application of the random forest algorithm to filter variables could avoid the increase in estimated parameters and insensitivity to outliers when dealing with multilayer categorical variables and is highly resistant to interference [29]. In addition, the nomogram lacks a complicated formula and instead predicts an individual’s risk of developing MAFLD on the basis of its scoring system and is therefore highly acceptable and can be effectively applied to the general population [30]. More importantly, our nomogram not only succinctly demonstrates the relationship between MAFLD and its risk factors but also facilitates identifying changes in prevalence in accordance with the changes in the values of specific risk factors. A growing body of evidence shows that nomograms can predict disease risk in a visual and understandable way [17].

Notably, 10 variables were included in our nomogram. The diagnostic criteria of MAFLD have been proven to be associated with five of these 10 factors, namely, BMI, WC, SBP, FPG, and TG, but not with sex, age, WHR, ALT, and UA. Our study innovatively included sex and WHR in the variable screening. Males are at a higher risk of developing MAFLD than females, as has been confirmed in many studies [31–33]. While sex cannot be modified, it can be used as a categorical indicator to advise the highly susceptible population of men to be screened for MAFLD. Although research on the use of WHR as an anthropometric indicator to predict MAFLD is limited, Zheng et al. [34] and Cai et al. [35] demonstrated that WHR has a high diagnostic value for NAFLD. In the present study, the multivariate logistic regression results indicated that abnormal WHR was strongly and positively associated with the risk of MAFLD (OR = 2.01, 95% CI: 1.93–2.10). This finding confirmed that WHR needs to be used as one of the routine indicators for predicting and screening MAFLD. Moreover, our study found that high ALT values were tightly associated with a high risk of MAFLD (OR = 2.39, 95% CI: 2.26–2.52), suggesting that the ALT biomarker is an important reference for screening MAFLD, although evidence showing that ALT values could be regarded as the diagnostic standard for NAFLD to some extent is controversial [36–38]. Consistent with a previous study [39, 40], the present work highlighted the importance of UA in predicting MAFLD (OR = 1.65, 95% CI 1.57–1.73). Thus, these identified parameters in our MAFLD-predictive model are not only easily available but also reliable and accurate.

To our knowledge, our nomogram is the first nomogram for predicting MAFLD risk that is applicable to the Chinese general population and may compensate for some of the shortcomings of previous MAFLD screening tools. For example, the nomogram for predicting the risk of MAFLD in overweight and obese populations reported by Song et al. is unavailable to the general population [14], the sensitivity and specificity of the clinical and laboratory nomogram (CLN) model for predicting NAFLD need improvement, and the CLN model is inapplicable to the newly defined condition of MAFLD [41]. The MAFLD prediction nomogram based on demography, laboratory factors, anthropometry, and comorbidities can well predict MAFLD but may be inappropriate for the Chinese population because the BMI and WC thresholds for the Asian population differ from the diagnostic criteria for the US population [15]. Specifically, Asians are defined as overweight/obese with lower cutoff values for BMI (BMI ≥ 23 kg/m^2^) and WC anomaly (WC ≥ 90/80 cm) compared to Caucasians [1]. Our nomogram could solve these problems because it was constructed on the basis of physical examination data related to MAFLD in the general Chinese population and has high sensitivity and specificity. Our nomogram not only could support clinicians in screening for MAFLD and determining whether participants need further abdominal ultrasound to confirm the diagnosis of MAFLD, it could also provide self-management to patients with MAFLD who are potentially at risk to seek timely medical assistance.

### Study strengths and limitations

The strengths of this study include the large sample of participants (138,664), which increases the reliability and statistical power of the nomogram. The combination of classical regression methods and the random forest algorithm ensured the best combination of factors included in the prediction model. More importantly, our prediction model can be widely applied to health management (physical examination) centers for rapid screening of MAFLD, and the presentation of the nomogram also makes it easy to assess the risk of MAFLD, which contributes to realizing graded management and timely referral of MAFLD, thereby improving the overall level of MAFLD prevention and treatment.

However, our study also has several potential limitations. First, in this study, the diagnosis of MAFLD was based on steatosis detected by liver ultrasonography rather than biopsy because performing liver biopsy in a large-scale survey was impractical. Future studies will add liver biopsy where possible to ensure the accuracy of MAFLD diagnosis. Second, the exclusion of some patients who underwent physical examination due to missing data may have led to some bias. Third, patients with MAFLD diagnosed by ultrasonography lacked data on 2-h postload glucose, HbA1c, HOMA-IR, and Hs-CRP.

## Conclusion

Our study used routine indicators to establish a risk-stratified nomogram that screens for the risk of MAFLD in the physical examination population. Clinicians can provide individualized plans to subjects in accordance with risk assessment. High-risk individuals, for whom early lifestyle interventions may help prevent disease progression and reduce the risk of adverse outcomes, should be referred for additional diagnostic testing to confirm NAFLD.

## Supplementary Information

**Additional file 1: Supplementary Fig. 1. **Subject selection flowchart. **Supplementary Fig. 2.** ROC curves for the prediction model: (a) the development dataset and (b) the validation dataset. **Supplementary Fig. 3. **Calibration curves for the nomogram for predicting MAFLD risk (a) the development dataset and (b) the validation dataset. Note: The black line indicates the perfect prediction of the ideal model and the dotted line indicates the performance of the nomogram. Calibration curves that are closer to the diagonal line have higher prediction accuracy. **Supplementary Table 1.** Predictors of MAFLD risk in the physical examination population.

## Data Availability

The datasets used and/or analyzed during the current study are available from the corresponding author on reasonable request.

## References

[1] Eslam M, Newsome PN, Sarin SK (2020). A new definition for metabolic dysfunction-associated fatty liver disease: An international expert consensus statement. J Hepatol.

[2] Eslam M, Sarin SK, Wong VW (2020). The Asian Pacific Association for the Study of the Liver clinical practice guidelines for the diagnosis and management of metabolic associated fatty liver disease. Hepatol Int.

[3] Lee H, Lee YH, Kim SU, Kim HC (2021). Metabolic Dysfunction-Associated Fatty Liver Disease and Incident Cardiovascular Disease Risk: A Nationwide Cohort Study. Clin Gastroenterol Hepatol.

[4] Sarin SK, Kumar M, Eslam M (2020). Liver diseases in the Asia-Pacific region: a Lancet Gastroenterology & Hepatology Commission. Lancet Gastroenterol Hepatol.

[5] Chan KE, Koh TJL, Tang ASP (2022). Global Prevalence and Clinical Characteristics of Metabolic-associated Fatty Liver Disease: A Meta-Analysis and Systematic Review of 10 739 607 Individuals. J Clin Endocrinol Metab.

[6] Liang Y, Chen H, Liu Y (2022). Association of MAFLD with diabetes, chronic kidney disease, and cardiovascular disease: a 4.6-year cohort study in China. J Clin Endocrinol Metab.

[7] Fan J, Luo S, Ye Y (2021). Prevalence and risk factors of metabolic associated fatty liver disease in the contemporary South China population. Nutr Metab (Lond).

[8] Chalasani N, Younossi Z, Lavine JE (2012). The diagnosis and management of non-alcoholic fatty liver disease: practice Guideline by the American Association for the Study of Liver Diseases, American College of Gastroenterology, and the American Gastroenterological Association. Hepatology.

[9] Hernaez R, Lazo M, Bonekamp S (2011). Diagnostic accuracy and reliability of ultrasonography for the detection of fatty liver: a meta-analysis. Hepatology.

[10] Carvalhana S, Leitão J, Alves AC, Bourbon M, Cortez-Pinto H (2014). How good is controlled attenuation parameter and fatty liver index for assessing liver steatosis in general population: correlation with ultrasound. Liver Int.

[11] Kotronen A, Peltonen M, Hakkarainen A (2009). Prediction of non-alcoholic fatty liver disease and liver fat using metabolic and genetic factors. Gastroenterology.

[12] Lee JH, Kim D, Kim HJ (2010). Hepatic steatosis index: a simple screening tool reflecting nonalcoholic fatty liver disease. Dig Liver Dis.

[13] Li L, You W, Ren W (2017). The ZJU index is a powerful index for identifying NAFLD in the general Chinese population. Acta Diabetol.

[14] Song D, Ge Q, Chen M (2022). Development and Validation of a Nomogram for Prediction of the Risk of MAFLD in an Overweight and Obese Population. J Clin Transl Hepatol.

[15] Zou H, Zhao F, Lv X, Ma X, Xie Y (2022). Development and validation of a new nomogram to screen for MAFLD. Lipids Health Dis.

[16] Park SY (2018). Nomogram: an analogue tool to deliver digital knowledge. J Thorac Cardiovasc Surg.

[17] Bonnett LJ, Snell KIE, Collins GS, Riley RD (2019). Guide to presenting clinical prediction models for use in clinical settings. BMJ..

[18] Moons KG, Altman DG, Reitsma JB (2015). Transparent Reporting of a multivariable prediction model for Individual Prognosis or Diagnosis (TRIPOD): explanation and elaboration. Ann Intern Med.

[19] World Health Organization. Regional Office for the Western, Pacific. The Asia-Pacific perspective: redefining obesity and its treatment. https://apps.who.int/iris/handle/10665/206936. Sydney: Health Communications Australia; 2000. Accessed 20 May 2020.

[20] American Diabetes Association (2016). Standards of medical care in diabetes—2016. Diabetes Care.

[21] Chinese Society of Endocrinology (2020). Guideline for the diagnosis and management of hyperuricemia and gout in China (2019). Chinese Journal of Endocrinology and Metabolism.

[22] Oliver NT, Hartman CM, Kramer JR, Chiao EY (2016). Statin drugs decrease progression to cirrhosis in HIV/hepatitis C virus coinfected individuals. AIDS.

[23] The Joint Committee on The Revision of Guidelines for the Prevention and Treatment of Dyslipidemia in Chines Adults. The guidelines for the prevention and treatment of dyslipidemia in Chinese Adults (2016 Revision). Chin Circ J. 2016;31(10):937–50; (in Chinese).

[24] Needleman L, Kurtz AB, Rifkin MD, Cooper HS, Pasto ME, Goldberg BB (1986). Sonography of diffuse benign liver disease: accuracy of pattern recognition and grading. AJR Am J Roentgenol.

[25] Breiman L (2001). Random forests. Mach Learn.

[26] Harrell FE, Lee KL, Califf RM, Pryor DB, Rosati RA (1984). Regression modelling strategies for improved prognostic prediction. Stat Med.

[27] Vickers AJ, Elkin EB (2006). Decision curve analysis: a novel method for evaluating prediction models. Med Decis Making.

[28] Bertot LC, Jeffrey GP, Wallace M (2017). Nonalcoholic fatty liver disease-related cirrhosis is commonly unrecognized and associated with hepatocellular carcinoma. Hepatol Commun.

[29] Lv J, Ren H, Guo X (2022). Nomogram predicting bullying victimization in adolescents. J Affect Disord.

[30] Iasonos A, Schrag D, Raj GV, Panageas KS (2008). How to build and interpret a nomogram for cancer prognosis. J Clin Oncol.

[31] Chen YL, Li H, Li S (2021). Prevalence of and risk factors for metabolic associated fatty liver disease in an urban population in China: a cross-sectional comparative study. BMC Gastroenterol.

[32] Li H, Guo M, An Z (2020). Prevalence and Risk Factors of Metabolic Associated Fatty Liver Disease in Xinxiang, China. Int J Environ Res Public Health.

[33] Yuan Q, Wang H, Gao P (2022). Prevalence and Risk Factors of Metabolic-Associated Fatty Liver Disease among 73,566 Individuals in Beijing, China. Int J Environ Res Public Health.

[34] Zheng RD, Chen ZR, Chen JN, Lu YH, Chen J (2012). Role of Body Mass Index, Waist-to-Height and Waist-to-Hip Ratio in Prediction of Nonalcoholic Fatty Liver Disease. Gastroenterol Res Pract.

[35] Cai J, Lin C, Lai S (2021). Waist-to-height ratio, an optimal anthropometric indicator for metabolic dysfunction associated fatty liver disease in the Western Chinese male population. Lipids Health Dis.

[36] Ma X, Liu S, Zhang J (2020). Proportion of NAFLD patients with normal ALT value in overall NAFLD patients: a systematic review and meta-analysis. BMC Gastroenterol.

[37] Martin-Rodriguez JL, Gonzalez-Cantero J, Gonzalez-Cantero A, Arrebola JP, Gonzalez-Calvin JL (2017). Diagnostic accuracy of serum alanine aminotransferase as biomarker for nonalcoholic fatty liver disease and insulin resistance in healthy subjects, using 3T MR spectroscopy. Medicine (Baltimore)..

[38] Verma S, Jensen D, Hart J, Mohanty SR (2013). Predictive value of ALT levels for non-alcoholic steatohepatitis (NASH) and advanced fibrosis in non-alcoholic fatty liver disease (NAFLD). Liver Int.

[39] He J, Ye J, Sun Y, Feng S, Chen Y, Zhong B (2022). The Additive Values of the Classification of Higher Serum Uric Acid Levels as a Diagnostic Criteria for Metabolic-Associated Fatty Liver Disease. Nutrients.

[40] Wan X, Xu C, Lin Y (2016). Uric acid regulates hepatic steatosis and insulin resistance through the NLRP3 inflammasome-dependent mechanism. J Hepatol.

[41] Cen C, Wang W, Yu S (2020). Development and validation of a clinical and laboratory-based nomogram to predict nonalcoholic fatty liver disease. Hepatol Int.

