# Supplementary material for: Development and validation of a nomogram for predicting metabolic-associated fatty liver disease in the Chinese physical examination population
Source: Lipids Health Dis. 2023 Jun 29;22:85. doi: 10.1186/s12944-023-01850-y (PMC10308730; doi:10.1186/s12944-023-01850-y)
Supplement: Supplementary file 1 — Additional file 1: Supplementary Fig. 1. Subject selection flowchart. Supplementary Fig. 2. ROC curves for the prediction model: (a) the development dataset and (b) the validation dataset. Supplementary Fig. 3. Calibration curves for the nomogram for predicting MAFLD risk (a) the development dataset and (b) the validation dataset. Note: The black line indicates the perfect prediction of the ideal model and the dotted line indicates the performance of the nomogram. Calibration curves that are closer to the diagonal line have higher prediction accuracy. Supplementary Table 1. Predictors of MAFLD risk in the physical examination population. [file 12944_2023_1850_MOESM1_ESM.doc]

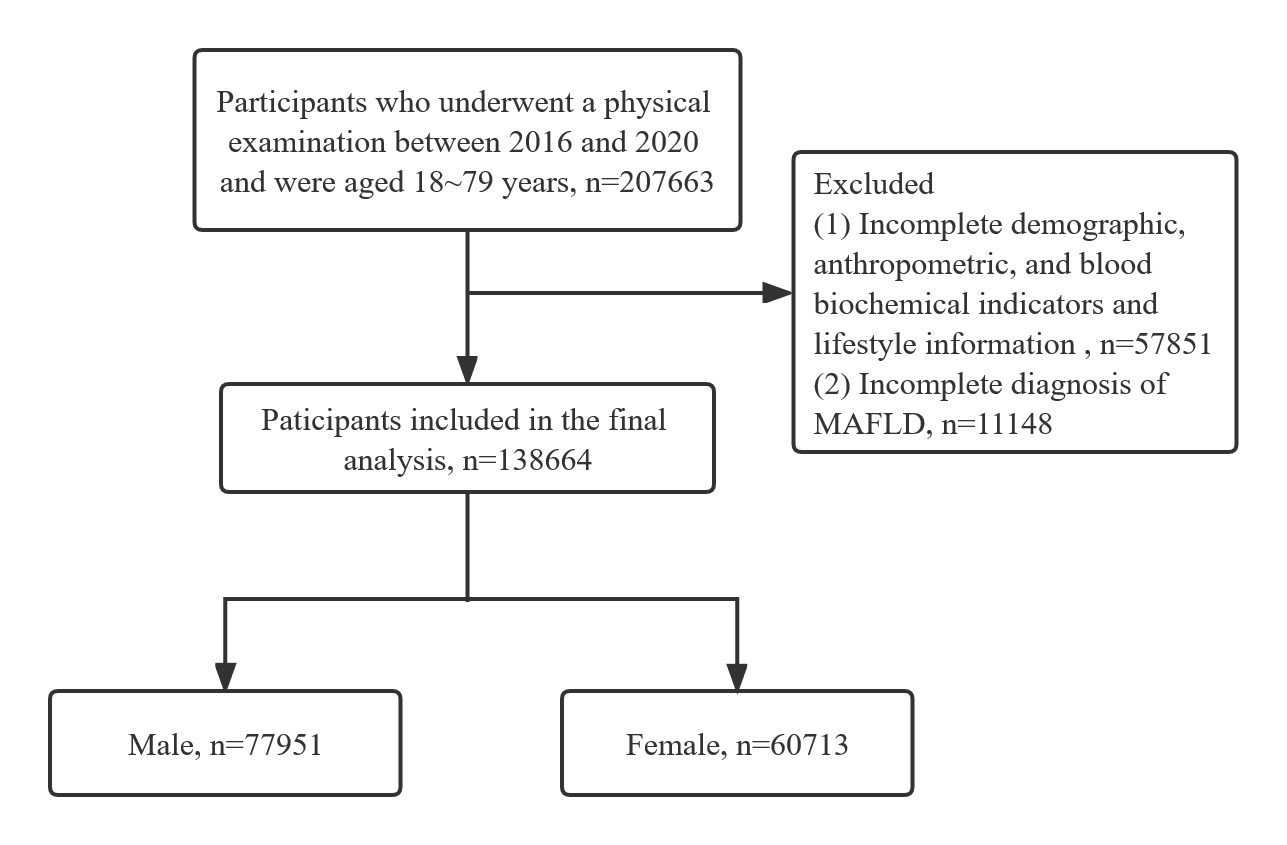


**Supplementary Fig.1 Subject selection flowchart**


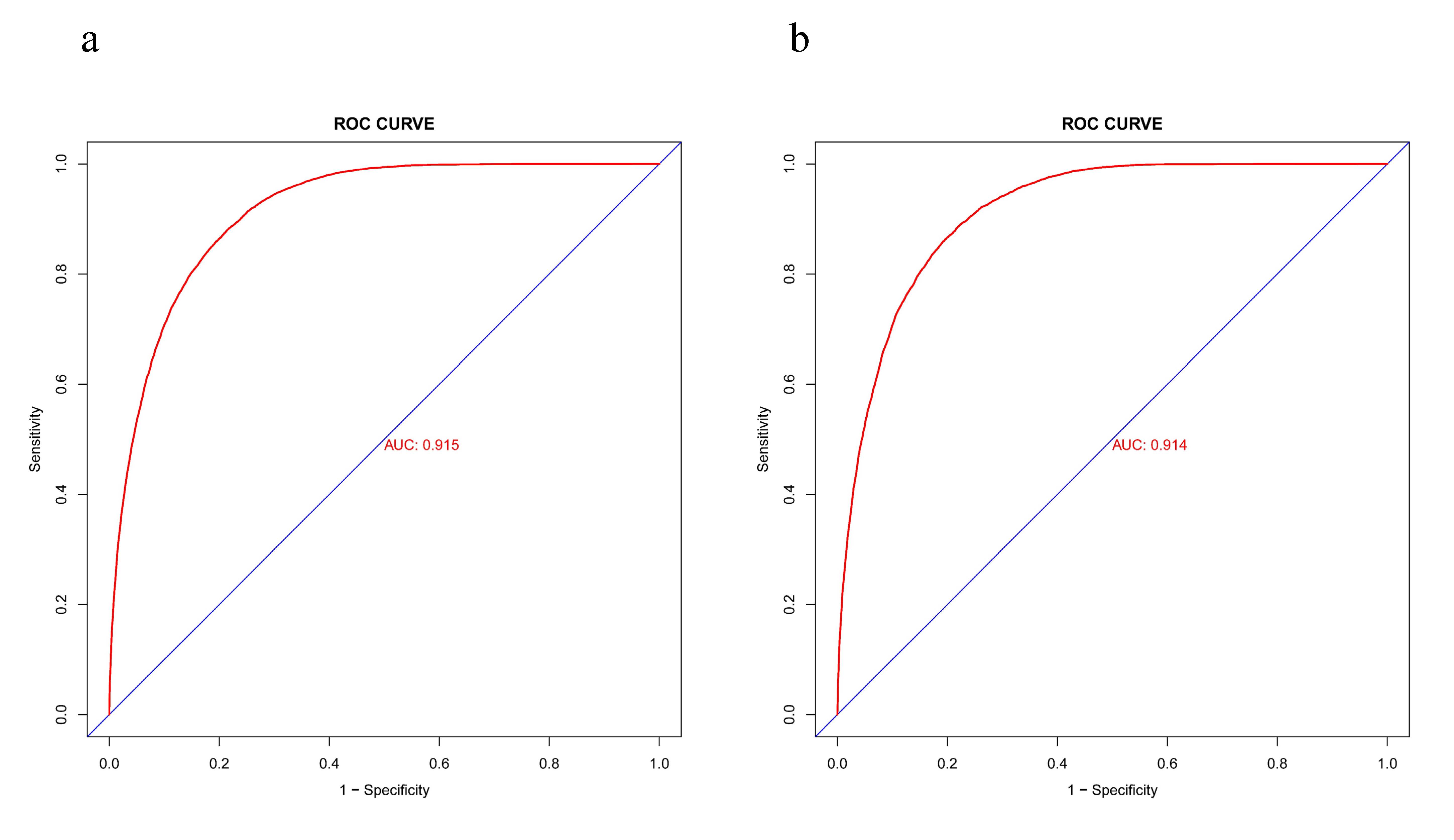


**Supplementary Fig. 2 ROC curves for the prediction model: (a) the development dataset** **and**

**(b) the validation dataset**


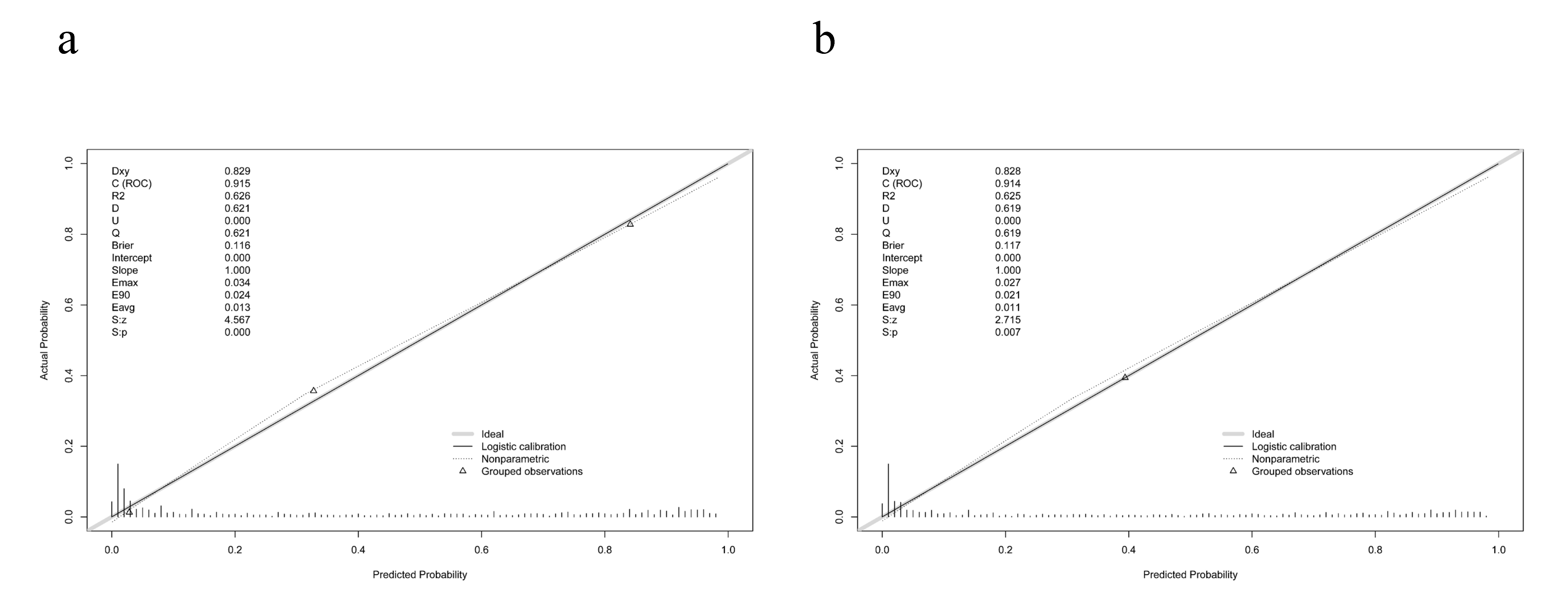


**Supplementary Fig. 3 Calibration curves for the nomogram for predicting MAFLD risk (a) the development dataset and (b) the validation dataset. Note:** The black line indicates the perfect prediction of the ideal model and the dotted line indicates the performance of the nomogram. Calibration curves that are closer to the diagonal line have higher prediction accuracy.


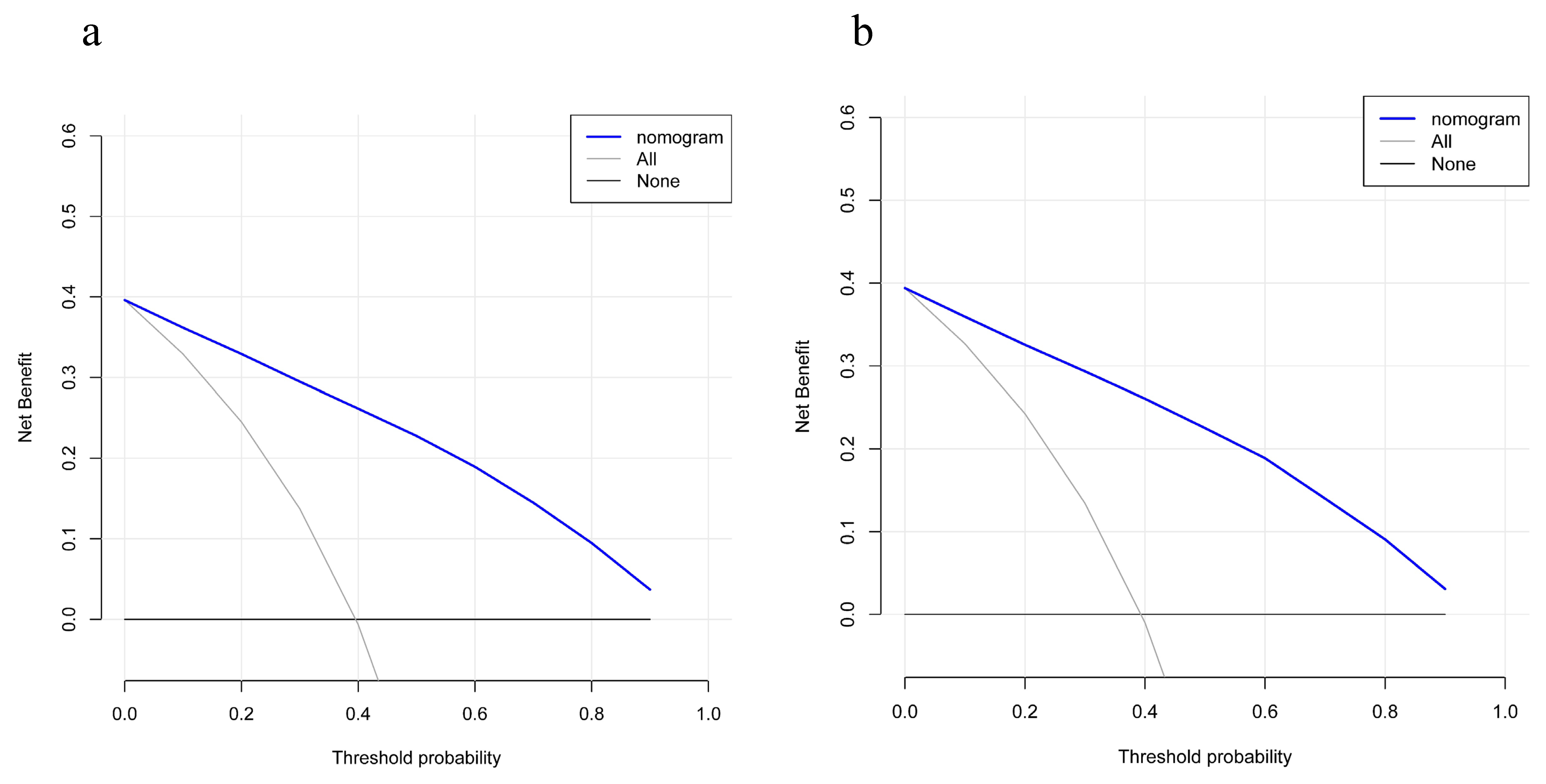


**Supplementary Fig. 4 Decision curves for the proposed nomogram model in (a)** **the development dataset and (b) the validation dataset. Note:** Black line: the net benefit is zero assuming no patients are treated. Gray line: all patients are assumed to be treated. Blue lines: if the proposed model exceeds the threshold, the patient would be treated.

**Supplementary Table 1 Predictors of MAFLD risk in the physical examination population**

| Predictors | | | | Options | | | | Score | | |
| --- | --- | --- | --- | --- | --- | --- | --- | --- | --- | --- |
| BMI (kg/m^2^) | | | | <18.50 | | | | 0 | | |
|  |  |  |  | 18.50–22.99 | | | | 45 | | |
|  |  |  |  | 23.00–24.99 | | | | 81 | | |
|  |  |  |  | ≥25.00 | | | | 100 | | |
| WC | | | | Normal | | | | 0 | | |
|  |  |  |  | Abnormal | | | | 15 | | |
| WHR | | | | Normal | | | | 0 | | |
|  |  |  |  | Abnormal | | | | 15 | | |
| TG (mmol/L) | | | | <1.7 | | | | 0 | | |
|  |  |  |  | ≥1.7 | | | | 24 | | |
| Sex | | | | Female | | | | 0 | | |
|  |  |  |  | Male | | | | 12 | | |
| ALT (U/L) | | | | ≤40 | | | | 0 | | |
|  |  |  |  | >40 | | | | 18 | | |
| FPG (mmol/L) | | | | <5.6 | | | | 0 | | |
|  |  |  |  | ≥5.6 | | | | 12 | | |
| Age (years) | | | | 18–29 | | | | 0 | | |
|  |  |  |  | 30–44 | | | | 9 | | |
|  |  |  |  | 45–59 | | | | 14 | | |
|  |  |  |  | 60–79 | | | | 13 | | |
| UA (μmol/L) | | | | ≤420 | | | | 0 | | |
|  |  |  |  | >420 | | | | 10 | | |
| SBP (mmHg) | | | | <130 | | | | 0 | | |
|  |  |  |  | ≥130 | | | | 6 | | |
|  | Risk prediction: |  |  | |  |  |  | |  |  |
|  | Total score, points | ≤94 | 95–122 | | 123–139 | 140–157 | 158–185 | | >185 |  |
|  | MAFLD risk, % | ≤10 | 11–30 | | 31–50 | 51–70 | 71–90 | | >90 |  |
